# The Impact of Hidradenitis Suppurativa on Health Care Decision Making and Vaccines

**DOI:** 10.21203/rs.3.rs-3778510/v1

**Published:** 2023-12-22

**Authors:** Najiba Afzal, Marjorie Archila, John S. Barbieri, Alexandra Charrow, Arash Mostaghimi, Lourdes Perez Chada, Megan H. Noe

**Affiliations:** University of California, Davis; Brigham and Women’s Hospital; Brigham and Women’s Hospital; Brigham and Women’s Hospital; Brigham and Women’s Hospital; Brigham and Women’s Hospital; Brigham and Women’s Hospital

## Abstract

**Background:**

Hidradenitis suppurativa (HS) is a chronic skin disease that causes significant burden for patients in multiple aspects of their life. However, the details regarding the impact on factors aside from skin are limited.

**Objective:**

We explored patient perspectives around the impact of HS on personal health and how that affects a patient’s health care decision making.

**Methods:**

Individual, semi-structured, virtual interviews were conducted with adults that have HS by a trained medical student. The interviews were performed over a private, video conference platform. English speaking individuals between the ages of 18–45 with a diagnosis of HS for at least 1 year were invited to participate in the study. The transcripts were coded by the medical student and a research assistant and discrepancies were resolved by group consensus. This study followed the reporting guidelines of the Consolidated Criteria for Reporting Qualitative Research.

**Results:**

23 participants were interviewed in which 21 participants (91%) were female and 2 participants (9%) were male. The mean age was 31.2 years. Patients expressed an increased awareness of their personal health because of their HS, including considering HS with respect to what they ate, the medications they took, the physicians they sought, and their family planning decision. Some participants stated that HS made them more likely to receive vaccines while others described the two are unrelated.

**Conclusions:**

Patients with HS considered their skin disease when making medical decisions broadly. Many specifically considered their disease when making decisions regarding health maintenance and immunizations though some did not consider the two related.

## Introduction

Hidradenitis Suppurativa (HS) is a chronic skin condition characterized by inflammatory nodules and abscesses that progress to draining sinus tracts, punctuated by periods of disease exacerbation and flaring. In addition to a decreased quality of life^1^, HS is often co-morbid with other high-risk conditions^2^ including cardiovascular disease, metabolic syndrome, and diabetes mellitus and is treated with higher doses of broad immunosuppressants than is typical for other skin conditions^3^. Given the increased morbidity of both HS and the treatments associated with it, an in-depth understanding is needed of patients’ perceptions of their disease in that context. Previous studies have emphasized the crucial need for vaccination, especially in immunosuppressed patients^4^; however, vaccine rates in patients with chronic inflammatory diseases remain low^5^. Vaccination in patients with HS is particularly important for a variety of indications including against HPV to limit progression of disease to squamous cell carcinoma^6^, Shingrex vaccine for potential herpes zoster risk with new JAK-inhibitors^7^, and Prevnar vaccine for treatment with TNF-alpha inhibitors^8^. Little is known about how patients come to make medical decisions about their HS and whether they consider their HS when making healthcare decisions especially regarding vaccination. In this study, we used systematic, in-depth qualitative interviews with patients with HS to explore patient perspectives of their disease, its impact on their personal health and its relationship to their health-care decision making process.

## Methods

### Study design

The qualitative approach used in this study was grounded theory. Individual semistructured, virtual interviews were performed with adults diagnosed with HS. The virtual interviews were conducted over a video conference platform. The interview questions explored patients’ perspectives on how their personal health and health care decision making are impacted by HS including the impact of HS around vaccine decision making. This study was deemed exempt (Category 2) by the Mass General Brigham IRB, and patients provided verbal consent before participating in the interview.

### Study participants

Invitations to participate in the research were sent to individuals between 18–45 years of age who had an appointment for hidradenitis suppurativa at Brigham and Women’s Hospital dermatology clinics between 1/1/2021–6/30/2022. English speaking individuals between the ages of 18–45 with a diagnosis of HS for at least 1 year were included in the study. Participants less than 18 years of age, deceased, or those who opted out of research were excluded from this study.

### Data collection

After careful review of literature in addition to the expertise from the investigators (MHN, APC, LPC, JSB,) an interview guide was created. Pilot testing of the interview guide was performed by stimulating 5 interviews after which feedback was considered and the guide was updated. A medical student research fellow who had received formal interview training and had no previous relationship to the study subjects conducted interviews from 10/27/2022 to 01/18/2023. Each interview was conducted virtually, in a private room, lasted 20–30 minutes and were audio-recorded. The audio-recorded interviews were then transcribed using institutionally secured Microsoft Word and edited for accuracy. Interviews with 23 subjects were necessary to achieve thematic saturation.

### Data analysis

The interview script development and data analyses were completed using guidelines from the Health Brief Model ^9^. Two senior investigators (MHN, APC) reviewed the five initial transcripts and created a codebook to identify major themes from the transcribed data in addition to the investigator’s baseline clinical knowledge. Each of the interview transcripts was independently coded in NVIVO by a medical student research fellow and research assistant (NA, MA). Each round of coding was followed by a group discussion and discrepancies were resolved by consensus. After major themes were identified, code frequencies were calculated and representative quotations from the interview scripts were extrapolated for each theme. This study adhered to the reporting guidelines in accordance with the Consolidated Criteria for Reporting Qualitative Research ^10^.

## Results

A total of 23 study participants with hidradenitis suppurativa were interviewed in this study in which 21 participants (91%) were female and 2 participants (9%) were male. The mean age of participants was 31.2 (SD: 6.9, range: 19–43) (Table 1).

### Impact of HS on Personal Health

Most participants described HS as posing a significant impediment to their physical health and wellness by imposing painful or inconvenient limitations (Table 2). For example, one participant explained that *“there have been times where I’ve wanted to exercise, or in the summer I wanted to go swimming, however, if I was having a really bad HS flare, then I wouldn’t want to do those things because it’s too painful.”* Many patients also described how HS impacted their mental health. One participant stated, *“It impacts my mental health for sure because it’s very painful [...]and every time I’m in that situation, I just want to cry because I feel powerless.”* Additionally, patients described experiences that negatively impacted their mental/emotional health due to poor self-esteem, shame, lack of control over body, fear, and changes in sexual health. One participant described *“it really affects my body image and self-image. It took me a long time to come to terms with the fact that HS is not my fault.”*

Many patients described how living with HS not only imposed limits both physically and psychology as outlined above but also increased their awareness of their own health generally. Patients described being more aware of their diet and exercise habits as they related to HS flaring, being aware of medication changes as they might relate to disease, being more vigilant about how they chose their physicians, and being more deliberate about family planning. For example, one participant described changing their diet to prevent a flare up: “*I’m trying to see if there’s a connection to what I eat or what I drink or the movements that I’m making and how I feel.”* One participant explained that *“I have been wanting to conceive but I am limited by a lot of medications and treatments for HS.”*

### Impact of HS on Medical Decision Making:

When asked about what resources are used to make healthcare decisions, participants expressed getting the opinion of their physicians, pharmacists, families/colleagues as well as their peers for information regarding health care decisions. One participant explained that their “*primary care physician or [their] dermatologist are the two doctors that [they] go [to] for any kind of medication-related decision*.” The internet including social media, internet forums, governmental-sponsored websites and dermatology-specific websites, like the HS Foundation, were other resources identified by participants.

Participants in this study were asked how HS impacted their decisions regarding vaccines and the responses were varied (Table 3). One participant mentioned being more cognizant of mild side effects of vaccines due to their already immunocompromised state from HS medications. The participant explained *“I don’t want [vaccines] to interact with the Humira, which is helping HS, but it’s making my entire body weaker.”* Other patients described that having HS makes them more likely to get a vaccine. *“I know that my immunity is compromised, and so, I am much more aware of and likely to take vaccines than a typical healthy person.”* Furthermore, some patients expressed that HS does not impact decisions made about vaccines.

When asked about the HPV vaccine, 74% of participants reported receiving the vaccine, 17% did not receive the HPV vaccine and 9% were unsure. Upon further exploring patients’ knowledge about the HPV vaccine, some expressed they were aware that the vaccine can “*prevent HPV and cancer”* while others described *“I honestly don’t know that much about it [HPV vaccine]. My mom had me do it when I was a kid.”*

## Discussion

The patient perspectives explored in this qualitative study revealed that HS has a significant impact on multiple aspects of a patient’s perceived personal health which influences their decisions around their medical care. HS patients describe considering their skin condition when deciding which medications to take, which foods to eat, and which medical providers to seek. Female participants also explained their hesitation around becoming pregnant due to the fear of disease flaring, medication side effects on pregnancy and breastfeeding due to the location of lesions around the nipples. Patients cited HS flare avoidance as a driver of their healthcare decisions regarding the above choices with respect to food, medication, and family planning.

Additionally, these same perspectives are reflected in HS patients’ choices around vaccines. Participants discussed how HS influences their attitudes around treatment-related immunosuppression when making medical decisions, including about immunization. HS patients have previously reported concerns such as fear of long-term consequences and side effects specifically about the COVID-19 vaccine.^11^ Some patients are more cautious of their HS because of the immunosuppression associated with its treatment, making them more likely to receive any vaccine, whereas others expressed concerns about certain side effects or worsening of chronic illnesses, including HS. Patients may hesitate around receiving vaccines due to their fear of causing an HS flare up which in turn affects their personal health due to the significant physical limitations and pain. Moreover, the treatment for HS with immunosuppressants such as Humira causes apprehension around vaccinations as patients fear vaccines may interact with their treatment and result in exacerbation of their condition. For these reasons, some patients avoid vaccines and are therefore at a higher risk of contracting vaccine preventable diseases.

Sources for information on vaccination and other healthcare related information was varied but healthcare providers were a common source for patients. However, because many HS patients live on average of 7–10 years with the condition before a diagnosis is made, their trust in the medical community is a barrier to understanding their condition.^12^ The decreased trust in the primary source of health information for HS patients can lead to a lack of adherence to immunizations, and medical advice further hindering their health care. It is important for medical providers to build trust and increase conversations around vaccines with their patients to address any concerns or misconceptions, while considering contextualizing such conditions within the framework of patients’ HS.

While this qualitative study expands on the existing literature, there are also some limitations. Participants in this study were not classified based on disease severity and therefore we are not able to differentiate how health-related quality of life is related to disease severity. Additionally, we interviewed participants from only one academic center in Massachusetts, a state with easily accessible state-run insurance and where most patients are therefore insured; therefore, the responses may not represent the experiences of unique patient populations across the United States. However, learning any patient perspective provides invaluable information to clinicians.

## Conclusion

In conclusion, this qualitative study found that HS has a significant impact a patient’s perception of their personal health and how this affects their medical decision making as well as their decisions around vaccines. These findings are important because they provide more detailed insight about the individual patient experience of living with a chronic disease which can help physicians provide individualized, patient-centered education and treatment.

## Figures and Tables

**Table 1:** Patient Characteristics

Characteristics	N (%)
**Age, mean (SD), y**	31.96 (6.9)
**Sex**	
*Female*	21 (91)
*Male*	2 (9)
**Total subjects 23**	

**Table 2:** Code Frequencies for Non-Vaccine Themes

Concept	Representative quotation	Total N = 23 N(%)
**Theme: Impact of HS on Personal Health**		
Increased awareness of personal health	*“Just making sure that any sort of thing I have to take in the future isn’t affected by the HS medication that I’m taking”*	*19 (83)*
Physical Limitation	*“It causes pain and prevents me from doing certain activities that I typically would do if I wasn’t having a flare up or if I didn’t have HS at all.”*	*16 (70)*
Mental, Emotional effects	*“The areas that are affected for me is my groin and buttocks and it can be embarrassing.”*	*13(57)*
Activities of Daily Living	*“The last episode I had was below my breast and it was so painful that I couldn’t wear a bra, or a T-shirt. Therefore, I basically had to stay home for four days with no clothes.”*	*12(52)*
Time	*“I do monthly infusions, so I have to plan my work schedule around it.”*	*5 (22)*
Fertility, pregnancy, family planning	*“HS affects the way I think about things like, having another child, because of where I get my HS flares at.”*	*4(17)*
Interpersonal relationships	*“I was about to potentially be sexually active with my first boyfriend and since I get flare ups in my groin, it was something that really bothered me.”*	*3 (13)*
**Theme: Healthcare Information Resources**		
Physicians (primary care, dermatologist, specialists)	*- "I talked to the dermatologist and to my OB about having HS”*	*21 (91)*
Internet	*“I do a little bit of research online to learn more about side effects. I also check online to find out other benefits.”*	*9 (39)*
Friends/Family/Colleagues	*“I have a lot of relatives in the medical field, so I’ll talk to them before doing certain things.”*	5 (22)
Pharmacists	*“If it’s a big deal medication that’s being added, I would say I used to talk to a pharmacist because I had a better relationship with them.”*	*5 (22)*
Primary research studies & Secondary research articles	*“I usually do my own research when a doctor suggests something, and by that, I mean actually reading published peer review papers on it, and then I make my own decision.”*	3 (13)
Insurance company	*“[My insurance company] puts things out also”*	1 (4)
Peers, patient support groups/advocacy organization	*“Friends and just the people I surround myself with too. I’ll usually listen to what they say about things.”*	*1 (4)*

**Table 3:** Code Frequencies for Vaccine Themes

**Theme: Benefits of Vaccination**		
Personal	*“I’m immunocompromised, so [vaccines] are incredibly important to me because if I catch something, it can be much more severe. it’s important to me to get vaccines to minimize any serious illness.”*	22 (97)
Societal	*“There’s of course the societal impact, for instance if I’m protecting myself with the vaccine, I’m preventing the spread of it to somebody else.”*	*15 (65)*
Fulfil Requirement	*“I know that some countries you can’t visit unless you have certain shots.”*	*2 (9)*
**Theme: Risk of Vaccination**		
Side effects	*“I know you can get some symptoms afterwards, but when I got the first booster, I had really bad symptoms, like, fever, sweats, and chills.”*	*11 (48)*
None/ unknown	*“I’m sure there are. I’m not too sure about the side effects. it’s not always foolproof, and I’m sure there are risks, but for me that’s just not something I’ve ever experienced.”*	*4 (17)*
Allergic Reactions	*“Less people have allergic reactions to [vaccines] and some of the variants”*	*4 (17)*
Uncertainty/ Unseen future risks	*“I think there’s not enough information that’s known about how it will react with people.”*	*3 (13)*
Causes new illness / Exacerbation of chronic diseases	*“Some people say that others may develop issues, like heart issues, I’ve heard about that too.”*	*2 (9)*
Lack of safety	*“I’ve heard lots of things regarding different vaccines. I haven’t done deeper research about that. So, I cannot tell that it’s 100% safe”*	*1 (4)*
**Theme: Barriers to Vaccination**		
Limited access to health care	*“I’m sure there are other economic barriers, like if you don’t have health insurance or aren’t regularly seeing a doctor, then that would probably be a barrier to it.”*	*17 (74)*
Misinformation	*“I think also I think there’s a lot of misinformation out there that is easily accessible for people”*	*10 (43)*
Limited availability / Access to vaccine	*“I think supply and demand. I know when the COVID shot first came out it was very hard to get one because so many people wanted it.”*	8 (35)
Distrust	*“Distrust of the health care either from a perspective of being in a community that has reason to distrust some medical things or being in a community that may have general distrust of government and regulations.”*	*3 (13)*
**Theme: Benefits of Vaccination**		
Religious/Social barriers/pressures	*“Religious exceptions for certain vaccines, but I donť know enough about individual types of religion that that would prohibit it.”*	*3 (13)*
Fear/concern of side effects	*“I would also say a fear of needles because that’s definitely come into play in the past with me.”*	*2 (9)*
Allergies	*“I feel like people with allergic reactions might have barriers.”*	*1 (4)*
**Theme: Impact of HS on Vaccine Decision Making**		
None	*“I don’t think about vaccines at all related to HS”*	11 (48)
More likely to get vaccines	*“I know that my immunity is compromised, and so, I am much more aware of and likely to take vaccines than a typical healthy person.”*	*6 (26)*
Vaccine may worsen/flare chronic diseases, including HS	*“I got the flu vaccine once and my HS erupted. I assume it’s because of my antibodies, like the immune deposits.”*	*4 (17)*
Increased susceptibility to illness	*“I don’t want it to interact with the Humira, which is helping my HS, but it’s making my entire body weaker. For example, I might feel a little headache or a little something after I take the vaccine.”*	*2 (9)*
**Theme: Knowledge about HPV vaccine**		
I do not know about the vaccine	*“I’ve heard of it and that’s it. I don’t know anything else. I didn’t get it.”*	16 (70)
I know about the vaccine	*“it can prevent HPV and cancer”*	*7 (30)*

## What is already known about this topic?

Hidradenitis suppurativa (HS) is an inflammatory skin disease which affects the intertriginous regions. Patients with HS are at an increased risk of co-morbid medical conditions in the setting of their disease and are commonly treated with immunomodulatory treatments. As some newer treatments promise greater response through broader immunosuppression, little is known about how patients with HS make medical decisions. (68 / 70)

## What does this study add?

In this qualitative study, HS patients were first prompted to describe how their disease impacted their physical and mental health. Through this lens, participants discussed how these impacts have in-turn affected their medical decision making. Patients obtained medical information from a wide variety of sources including physicians, the internet, friends, family, and pharmacy staff. Patients considered their HS and treatment-related immunosuppression when making medical decisions, including about immunization. (68/70)

## Clinical Implication:

HS patients largely consider their skin disease when making decisions around their health. As HS is a condition with considerable co-morbidities and broad immunosuppressive regimens, it is important to be aware of these patient perspectives when counseling patients regarding medical decisions including health maintenance and vaccination. (55/70)
